# Contextual Factors Associated with Burnout among Chinese Primary Care Providers: A Multilevel Analysis

**DOI:** 10.3390/ijerph16193555

**Published:** 2019-09-23

**Authors:** Huiwen Li, Beibei Yuan, Qingyue Meng, Ichiro Kawachi

**Affiliations:** 1 School of Public Health, Peking University, Beijing 100191, China; 2 China Center for Health Development Studies, Peking University, Beijing 100191, China; 3 Department of Social and Behavioral Sciences, Harvard T.H. Chan School of Public Health, Boston, MA 02115, USA

**Keywords:** burnout, primary care providers, multilevel analysis, work environment

## Abstract

Burnout is a common and growing phenomenon in the health care setting. The objective of the present study is to examine contextual factors in the workplace associated with burnout among primary care providers (PCPs) in Shandong Province, China. A cross-sectional survey was conducted among 951 PCPs nested within 48 primary health institutions (PHIs). Burnout was measured using the Maslach Burnout Inventory–Human Services Survey (MBI–HSS). We used two-level random intercept linear regression models to examine individual- versus workplace-level risk factors for burnout. The result revealed that 33.12%, 8.83% and 41.43% PCPs were experiencing a high degree of emotional exhaustion (EE), depersonalization (DP) and low personal accomplishment (PA). In multilevel analysis, the most significant and common individual-level predictors of burnout were lack of perceived work support and autonomy. At the institutional level, workload was positively related to EE (odds ratio (OR): 6.59; 95% confidence interval (CI): 3.46–9.72), while work support was related to higher PA (OR: 3.49; 95% CI: 0.81–6.17). Greater attention should be paid to the influence of the work environment factors (workload and work support) to prevent burnout. Strategies such as increasing human resources allocated to PHIs and establishing a supportive work environment are encouraged to prevent and reduce burnout among PCPs in China.

## 1. Introduction

Strengthening the primary health care (PHC) system has been an essential pillar of China’s new health-care reform initiated in 2009 [1]. Over the past decade, China has sought to expand the primary care workforce, resulting in an increase in the number of primary care providers (PCPs) from 3.3 million in 2010 to 3.8 million in 2017. During the same period, however, the proportion of PCPs among all health workers declined from 40.0% to 32.6% [2]. The shortage of qualified PCPs remains one of the critical challenges in healthcare, hampering the further development of the PHC system [3,4]. Given the extended time it takes to train qualified health workers, there is great interest in strategies that can be implemented quickly to reduce burnout and improve the productivity of PCPs.

Burnout was first described by Herbert Freudenberger [5], and further conceptualized along three dimensions consisting of emotional exhaustion (EE), feelings of depersonalization (DP) and low personal accomplishment (PA) by Maslach and Jackson [6]. EE refers to feelings of being overextended and depleted of one’s emotional and physical resources. DP represents the interpersonal dimension of burnout and refers to the absence of feeling (i.e., an impersonal response) toward the recipients of one’s service. Lastly, reduced PA represents the self-evaluation dimension of burnout and refers to feelings of incompetence, as well as lack of value and personal effectiveness at work [7]. 

Burnout has been described as an epidemic among healthcare workers in the US and many other countries [8]. The problem is also common among doctors in China with an overall prevalence ranging from 66.5% to 87.8% [9]. Physician and staff burnout matters because it not only has negative impacts on their well-being and safety [10,11], but also affects the quality of healthcare provided [12,13,14]. For China’s health-care reform to achieve its goal of providing all citizens with affordable and equitable basic health care by 2020 [1], a better understanding is warranted of the factors that may influence burnout. With the huge population base and increasing demand for health care, Chinese doctors are experiencing tremendous workload especially for those who work in tertiary hospitals. One of the major reasons for high burnout among doctors in hospitals is the weak primary healthcare system, resulting in more patients losing trust in PCPs and seeking more specialized consultations in hospitals [15]. Doctors in primary health institutions (PHIs) are also facing great pressure in providing high-quality health services and the introduction of regular public health evaluation with limited healthcare resources, so that the prevalence of burnout is high in both hospitals and PHIs. However, there is a paucity of research exploring the factors related to burnout among PCPs.

Systematic reviews of burnout [9,16,17] have revealed contributors for physician burnout, including: (1) *personal* characteristics such as female gender, younger age, being unmarried, and higher education; (2) *job* characteristics such as lower seniority, excessive workload, lack of work support and work autonomy. That is, the primary focus of most studies has been directed toward individual characteristics that predict burnout, as well as more proximal job characteristics [18,19,20]. Evidence-based interventions to reduce physician burnout have tended to focus on what individuals can do for themselves, such as mindfulness training and assertiveness training. It has been argued that incorporating a *multilevel* perspective into burnout research could add valuable new insights and be more effective than focusing only at the individual level [21]. 

As most decisions affecting the workplace environment for healthcare are made at the institutional level, it is important to pay more attention to the shared experiences of health workers in particular institutions or hospitals. To date, only a few studies have identified how factors at the institutional level may related to burnout and found significant relationships between unit-level favorable perceptions of work environment dynamics (e.g. doctor–nurse collegial relations, good leadership and support) and nurses’ burnout experiences [22,23,24]. 

Previous studies from China have been more focused on describing the personal demographic factors and/or job characteristics that are associated with physician burnout, especially among physicians and nurses in specific hospital departments [25,26,27]. By contrast, the role of contextual factors in the workplace for burnout among PCPs has received far less attention. Efforts to identify the prevalence of burnout and its associated contextual factors among PCPs are therefore urgently needed. Our study therefore sought to address this gap and providing practicable information for health administrators to mitigate burnout in the primary practice setting. 

In the present study, we sought to assess the prevalence of burnout among PCPs working in public health institutions in one province of China, and to examine the multi-level risk factors associated with burnout.

## 2. Materials and Methods

### 2.1. Study Units and Participants

A cross-sectional study was conducted in three counties of Shandong province in Northeastern China. We chose Shandong as the study site because of the known geographic variations in economic development and population. In 2017, Shandong had a population of roughly 100 million, ranking second in China, and the largest number of PCPs (325,310) [2]. PCPs in China provide both generalist outpatient and inpatient care services, as well as basic public health services such as periodical health check-ups for elderly people. A multi-stage cluster sampling method was applied in this study. First, we selected three counties reflecting different levels of economic development. Second, all the PHIs were sampled in each county, for a total of 48 institutions. Finally, all PCPs including doctors, nurses and public health workers present on the day of the survey in the selected PHIs were invited to participate. This study was approved by the Ethics Committee of Peking University of Health Science Center (code of ethics: PKU201412128). Written informed consent from PCPs was obtained before they filled in the questionnaire. 

### 2.2. Measurement of Burnout

The Maslach Burnout Inventory (MBI) continues to be most widely used instrument to assess burnout [7,8]. Burnout was measured using the 22-item Chinese version of Maslach Burnout Inventory–Human Service Survey (MBI–HSS) questionnaires [28]. The 22 items are divided into three subscales: (1) emotional exhaustion (9 items), (2) depersonalization (5 items), and (3) personal accomplishment (8 items). Example items for EE, DP and PA are “I feel emotionally drained from my work”, “I feel I treat some recipients as if they were impersonal objects” and “I deal very effectively with the problems of my recipients”, respectively. All items are rated on a 7-point Likert scale ranging from 0 = never to 6 = daily. The internal consistency of the scale was tested using Cronbach’s alpha coefficient (α = 0.84 for EE, α = 0.73 for DP, and α = 0.86 for PA). Prior research has found that this scale demonstrated a high level of internal consistency, with a Cronbach’s alpha of 0.89 for EE, 0.79 for DP, and 0.87 for PA [28]. The cut-offs applied to define cases were EE: low ≤ 16; moderate 17–26; high ≥ 27; DP: low ≤ 6; moderate 7–12; high ≥ 13; and reduced PA: low ≥ 39; moderate 32–38; high ≤ 31 [6]. In multilevel analysis, we used the continuous scores for the burnout scales.

### 2.3. Measurement of Individual-Level and Institution-Level Variables

*Perceived workload* was assessed with one question: How would you rate your workload? Respondents could indicate their response on a 5-point, Likert-type scale ranging from 1 (very little) to 5 (very heavy). 

*Perceived work support* was assessed by one item: “Could you receive assistance from your department and personnel if you encounter difficulties or problems at work?” A 5-point Likert-type scale was used, scored 1–5 (‘hardly ever’, ‘little’, ‘sometimes’, ‘most of the time’ and ‘almost every day’). 

*Perceived work autonomy* was assessed by one item: “Do you have the autonomy to make decisions and actions in your workplace, such as how to structure your schedule? Respondents indicated their degree of agreement with the statement on a 5-point Likert-type scale ranging from 1 (hardly ever) to 5 (almost every day) with higher scores representing greater autonomy. 

Consistent with existing studies of organizational climate [29,30], individuals’ reports on perceived workload, work support and work autonomy were centered to their respective grand means [31] and included in the multilevel model at the institution level in order to capture each PHI’s workplace environment. 

Other control variables included gender (male female), age (<30, 30−39, ≥40 years), education (high-school graduate or below, some college, bachelor’s degree or above), employment type (temporary employee, long-term employee), years of experience (<5, 5−10, 11−20, ≥21), work role (prevention, clinical, clinical and prevention) and monthly salary (<4000, ≥4000).

### 2.4. Statistical Analysis

The hierarchical structure of the sample included two levels with PCPs at level 1 and institutions at level 2. A series of two-level random intercept linear regression models [32] were constructed to account for the survey design and describe the individual- and institutional-level variance in burnout (Model 1 to 2). Variance partition coefficient (VPC) was used to estimate the proportion of total variance in burnout scores attributable to the work unit. We specified the following basic model:

(1) $$B_{ij}=\beta_{0}+\beta_{1}\left(W_{ij}-W_{j}\right)+\beta_{2}W_{j}+\beta_{3}X_{ij}+\mu_{j}+\varepsilon_{ij}$$

where *B* is the relevant dependent variable for individual i (level 1) in institution j (level 2), *W* is the set of workplace environment variables (i.e., workload, work support and work autonomy) measured at the individual and institution levels, *X* is a vector of standard control variables (gender, age, marital status, educational level as demographic characteristics; employment type, years of experience, work role, monthly salary as job characteristics). We include the county as a fixed effect to control for unobserved heterogeneity across counties. The β’s are the “fixed” parameters to be estimated, whereas $\mu_{j}$ is the individual-specific random effect, and $\varepsilon_{ij}$ is the random component of the error term. 

By specifying the individual perception of their workplace environment as a ‘group-centered’ measure (i.e. $W_{ij}-W_{j}$), we removed the collinearity between the individual perception ($W_{ij}$) and the workplace analog ($W_{j}$). For example, if $W_{ij}$ is the individual’s perception of work support, $W_{j}$ is the aggregated level of work support (obtained by averaging the responses of all individuals belonging to the same institution) [33]. $\beta_{1}$ is interpreted as the association between individual-level workplace environment variables above and beyond any institution-level association and is hypothesized to be positive. Equation (1) can be re-expressed as:

(2) $$B_{ij}=\beta_{0}+\beta_{1}W_{ij}+\left(\beta_{2}-\beta_{1}\right)W_{j}+\beta_{3}X_{ij}+\mu_{j}+\varepsilon_{ij}$$

where $\beta_{2}-\beta_{1}$ is interpreted as the effect of individual-level workplace environment variables. Testing whether $\beta_{2}$ is statistically different from $\beta_{1}$ is, therefore, equivalent to testing whether institution-level workplace environment variables are independently associated with burnout above and beyond associations at the individual level.

For each of the three dimensions of burnout, we specified two separate models: model 1 includes individual-level factors and model 2 adds institution-level factors to model 1. Data were analyzed using STATA 14.0 for Windows. All *p* values were two sided with a *p* value less than 0.05 considered as statistically significant.

## 3. Results

### 3.1. Participant Characteristics

Among the 1148 PCPs invited to participate in the survey, 951 PCPs from 48 PHIs responded and were included in the present analysis. The demographic and job characteristics are summarized in Table 1. Overall, the participants were predominantly female (65.1%), married (90.5%), and temporary (75.4%) health workers. Only 16.1% of the PCPs were under 30 years old, and 39.6% received bachelor’s degree or above. Approximately 31.4% of the PCPs worked in this PHI more than 21 years, and the monthly salary on average was ¥4069 ($580). The largest occupational group was PCPs who only perform clinical work (45.0%), leaving 27.3% PCPs doing prevention work and 27.7% doing both clinical and prevention work. Also, the mean perceived workload, work support and work autonomy were 3.7 (standard deviation (SD) = 0.7), 3.8 (SD = 1.1) and 3.3 (SD = 1.1), respectively. 

### 3.2. Prevalence of Burnout

From the validated MBI–HSS questionnaire, the mean scores of EE, DP and PA were 21.87, 4.75 and 33.14, respectively, representing moderate, low and moderate level of burnout (Table 2). The overall prevalence of high EE (≥27 points) was 33.12% (n = 315), 8.83% (n = 84) had high DP (≥13 points), and 41.43% (n = 394) had low PA (≤31 points). 32.91% (n = 313), 19.77% (n = 188), and 20.50% (n = 195) PCPs experienced moderate level of EE, DP and reduced PA.

### 3.3. Factors Associated with Burnout Symptoms

In the empty (null) model of multilevel regression (without any explanatory variables included), we assessed the variance attributed to between-institutions variation. The VPCs of EE, DP and PA were 7.77%, 5.32% and 10.04%, respectively, indicating the extent to which the variance in burnout can be attributed to differences between institutions, and thus provided further justification to the use of multilevel analysis. 

Multilevel analysis of risk factors associated with burnout symptoms are presented in Table 3. At the individual level, male gender, workers with higher education level and only dealing with clinical work were more likely to have higher EE. Men were more likely to have DP simultaneously. However, workers who only deal with clinical work were less likely to have lower PA than those who only do prevention work.

Among other individual-level factors, both perceived work support and autonomy were significantly protectively associated with all three dimensions of burnout with similar magnitude of the effect estimates in both Model 1 and Model 2 (EE: coefficient = −1.72 vs. −1.73, *p* < 0.001; DP: coefficient = −0.45 vs. −0.46, *p* < 0.05; PA: coefficient = 2.23 vs. 2.27, *p* < 0.001 and EE: coefficient = −0.68 vs. −0.68, *p* < 0.05; DP: coefficient = −0.42 vs. −0.42, *p* < 0.05; PA: coefficient = 1.23 vs. 1.21, *p* < 0.01, respectively).

In terms of the institution level variables, workload was found to be positively associated with EE at both individual level (coefficient = 5.28, *p* < 0.001) and institutional level (coefficient = 6.59, *p* < 0.001). Inclusion of institution-level workload slightly reduced the magnitude of the effect estimates, showing that workload affects EE partly through “workload in the institutional setting”, shared by all PCPs who are susceptible to be affected by working in the same environment (coefficient = 5.30 vs. 5.28, *p* < 0.001). Given these findings, we can consider that PHIs in which PCPs collectively perceived heavy workload are more likely to transmit a sense of being overwhelmed and experiencing a negative work climate.

Within an institution, higher work support was associated with higher PA scores (coefficient = 3.49, *p* < 0.05), indicating that over and above the individual-level association, there was a stronger contextual association: an individual reporting a given level of support will, on average, feel more work support if he or she works in an institution that gives higher support to its workers. By contrast, work autonomy at the institutional level was not significantly associated with any dimension of burnout incorporating (Model 2). These findings suggest that work support was a somewhat more important workplace contextual determinant of burnout than institutional work autonomy. We checked for potential heterogeneity of estimated associations, but did not find any significant cross-level interactions between contextual exposures and gender/age.

## 4. Discussion

Burnout is prevalent among health care providers in high-income countries, as well as low and middle-income countries (LMICs) [34]. This study identified 33.12%, 8.83% and 41.43% of the participants as experiencing severe burnout in the domains of Emotional Exhaustion, Depersonalization and low Personal Accomplishment, respectively. These numbers are significantly higher than rates previously reported in China (EE: 13.4%; DP: 5.6%; reduced PA: 25.5%) [28]. The prevalence of burnout due to EE and DP in this study were lower compared to family doctors in high-income countries (EE: range 43% to 47.9%; DP: 35.2% to 46.3%), while the PA dimension was higher compared to studies conducted in western settings (17.4% to 32%) [35,36]. One possible explanation is that Chinese patients prefer to bypass the primary health-care system to seek better treatment in hospitals for better treatment, hence giving rise to a diminished sense of personal effectiveness (PA) among Chinese primary care providers. Meantime, emotional exhaustion arises from a combination of work overload and role conflict [7]. On the other hand, PCPs in China appear to care about their patients, as suggested by their much lower prevalence of depersonalization. This possibility highlights the importance of focusing on improving the contributors to EE and reduced PA as a means of mitigating burnout in primary care practice.

As expected, PCPs who experience higher workload had elevated EE scores while their DP and PA scores were not significantly different from each other, which is in line with a previous study that reported harmful effects of excessive workload in the domain of EE [26,37]. A previous multi-center study found that Chinese PCPs were especially overworked, working on average 46.6 ± 9.1 hours per week and servicing 15.3 ± 13.2 patients per day [15], resulting in inadequate physician–patient communication and increased medical errors. The implement of the National Basic Public Health Service Program (NBPHSP)—which made PCPs responsible for providing a package of 13 basic public health services for all residents—dramatically increased their workload because of limit workforce numbers and strict assessment standards. The foregoing evidence suggests that institutional decision makers need to consider structural reforms to solve the shortage of PCPs and associated burnout for the safety of both patients and physicians. Workload is an important variable that is amenable to changes in organizational policies. Thus, changing institutional policies is likely to remain the most effective strategy for lowering the prevalence of burnout. It is essential for future planning regarding the recruitment, training and appointment of additional PCPs in order to meet the demand for and availability of PCPs [38]. Government also needs to provide better career advancement opportunities and working conditions to appeal to more providers entering the PHI workforce, optimize the assessment standards of NBPHSP to reduce public health workload, and further integrate medical treatment with prevention activities.

According to the multilevel regression results of our data, the most significant and common determinant across all three burnout dimensions was perceived work support at the individual level. Consistent with previous results that perceived work support was a significant protective factor against burnout [18,26,39], we found that the more support the PCPs perceived, the less burnout they experienced. Our multilevel study also showed a relationship between the *average* level of social support within the institution and reduced PA, but no relationship between support and EE or DP. A supportive work climate helps to prevent burnout. Our findings can be explained by the job demands–resources (JD-R) model [40], which posits that interpersonal job resources (such as a supportive work climate) may act as a *buffer* in the relationship between job demands and job burnout [41]. Support from managers and coworkers can be considered an expression of a positive social support system and plays an important role in protecting workers from low PA. Thus, our results suggest that building a supportive work environment in PHIs—i.e., efforts to build the “social capital” of an organization (for example, by strengthening collaborative team-based integrated primary health care delivery)—may represent a crucial strategy in reducing low PA risk. In contrast to our findings, Constanze et al. found that at the institutional level, support for providers reduced the risk for EE and DP without controlling for perceived work support at the individual level [22]. This possibility highlights the importance of developing measures appropriate to local conditions. 

Perceived work autonomy was a significant positive contributing factor to reduced burnout in all dimensions, especially a low sense of personal accomplishment. The introduction of an essential drug list for PHIs has been shown to be correlated with an unintended loss of autonomy [42], because the list may be too restrictive for daily treatment needs. With more patients bypassing PHIs to seek care at hospitals, this situation may contribute to the sense of low personal effectiveness among PCPs. Interestingly, our study did not confirm an association between institution-level work autonomy and burnout. 

In terms of risk factors for burnout at the individual level, our findings corroborate previous studies [43] indicating higher scores among men. The male/female difference has been attributed to gender differences in stress coping, i.e. women are more likely to express themselves and release negative emotions than men. Consistent with prior findings [44,45], we also find that PCPs with higher education level were more likely to experience EE because of their higher personal and social expectations. Medical specialties vary widely with respect to work circumstances that affect the relative prevalence of burnout. In our study, doctors and nurses who performed only clinical work at PHIs were more likely to experience EE, but less likely to express DP compared to public health workers who are only engaged in prevention work. Moreover, there was no difference in burnout rates comparing PCPs who perform both preventive and clinical duties versus public health workers, which suggests that strategies that promote the combining preventive and clinical practices may alleviate EE among clinical workers and improve PA among public health workers.

Our study has several limitations. Firstly, the cross-sectional nature of this study limits our ability to draw casual inferences. We cannot establish temporal ordering between exposures and outcomes. Secondly, although we controlled for employee wages and work history as control variables, we cannot completely rule out the possibility of residual confounding by unobserved variables that lead to the sorting of different individuals into different working conditions (endogeneity and selection bias). Some additional variables that could determine such selection (which we lacked in our data) include employee personality and sickness absence history. Thirdly, our study relied on self-report measures of both working conditions (e.g., perceived workload) and burnout, thereby leading to the potential for common method bias. On the other hand, our institutional-level exposures were calculated by averaging the responses of all workers, thereby reducing the likelihood of a biased individual (or outlier) influencing the results.

## 5. Conclusions

The current study sought to identify the personal and institutional determinants of burnout among primary care providers in one province of China. We identified a high rate of EE and reduced PA in our sample. Our multilevel analysis demonstrated that EE was positively correlated with the institutional-level workload of PHIs, while low institutional-level work support was correlated with low PA, net of personal characteristics. These results point to the potential importance of structural (i.e., institutional) policies to mitigate burnout. 

On the individual level, factors such as perceived work support and autonomy have strong relationships with all three burnout dimensions, suggesting that strategies that improve perceived work support and autonomy may alleviate job burnout among PCPs. Our multilevel perspective provided insights into a broader set of contextual factors that are associated with burnout as well as potential guidance for healthy workplace promotion. Strategies such as establishing a supportive work environment with a more manageable workload may be effective methods for burnout prevention and management among PCPs in China. Extending preventive measures, such as strengthening teamwork cooperation, time-management strategies and communication skills training are also suggested for individuals.

## Figures and Tables

**Table 1:** Demographic and job characteristics of participants (n = 951).

Covariate	n (%)
**Demographic characteristics**	
Gender	
Male	332 (34.9)
Female	619 (65.1)
Age group (years)	
<30	153 (16.1)
30−39	401 (42.2)
≥40	397 (41.7)
Education	
High-school graduate or blow	178 (18.7)
Some college	396 (41.6)
Bachelor’s degree or above	377 (39.6)
Marital status	
Married	861 (90.5)
Single/divorced/widowed	90 (9.5)
**Job characteristics**	
Employment type	
Temporary employee	717 (75.4)
Long-term employee	234 (24.6)
Years of experience	
<5	203 (21.4)
5−10	217 (22.8)
11−20	232 (24.4)
≥21	299 (31.4)
Work role	
Prevention	260 (27.3)
Clinical	428 (45.0)
Clinical and prevention	263 (27.7)
Monthly salary	
<4000	422 (44.4)
≥4000	529 (55.6)
Perceived workload, mean (SD)	3.7 (0.7)
Perceived work support, mean (SD)	3.8 (1.1)
Perceived work autonomy, mean (SD)	3.3 (1.1)

**Table 2:** Burnout symptoms in primary care providers (n = 951).

	Score Mean ± SD	Degree		
		Low n (%)	Moderate n (%)	High n (%)
EE	21.87 ± 10.71	323 (33.96)	313 (32.91)	315 (33.12)
DP	4.75 ± 5.10	679 (71.40)	188 (19.77)	84 (8.83)
Reduced PA	33.14 ± 10.81	362 (38.07)	195 (20.50)	394 (41.43)

Note: emotional exhaustion (EE), depersonalization (DP) and personal accomplishment (PA).

**Table 3:** Multilevel linear regression analysis of factors related to burnout.

Variables	Model 1 Coefficient (95% CI)			Model 2 Coefficient (95% CI)		
	EE	DP	PA	EE	DP	PA
**Individual level**						
Gender						
Male	1 (reference)	1 (reference)	1 (reference)	1 (reference)	1 (reference)	1 (reference)
Female	−1.52 (−2.85, −0.20) *	−1.17 (−1.86, −0.48) **	0.87 (−0.51, 2.26)	−1.53 (−2.86, −0.20) *	−1.20 (−1.90, −0.51) **	0.99 (−0.40, 2.37)
Age group (years)						
<30	1 (reference)	1 (reference)	1 (reference)	1 (reference)	1 (reference)	1 (reference)
30−39	0.27 (−1.93, 2.48)	0.12 (−1.03, 1.27)	−0.96 (−3.27, 1.35)	0.15 (−2.05, 2.36)	0.03 (−1.13, 1.18)	−0.71 (−3.01, 1.59)
≥40	−0.10 (−2.75, 2.56)	−0.25 (−1.64, 1.14)	−0.78 (−3.55, 2.00)	−0.31 (−2.96, 2.35)	−0.31 (−1.71, 1.08)	−0.68 (−3.45, 2.10)
Education						
High-school graduate or blow	1 (reference)	1 (reference)	1 (reference)	1 (reference)	1 (reference)	1 (reference)
Some college	2.28 (0.49, 4.06) *	0.02 (−0.91, 0.95)	1.19 (−0.68, 3.06)	2.59 (0.80, 4.38) **	0.03 (−0.90, 0.97)	1.15 (−0.71, 3.02)
Bachelor’s degree or above	2.77 (0.87, 4.67) **	−0.26 (−1.25, 0.72)	1.25 (−0.74, 3.24)	3.12 (1.21, 5.03) **	−0.21 (−1.21, 0.79)	1.08 (−0.91, 3.08)
Marital status						
Single/divorced/widowed	1 (reference)	1 (reference)	1 (reference)	1 (reference)	1 (reference)	1 (reference)
Married	−0.51 (−2.84, 1.82)	0.28 (−0.94, 1.49)	−0.50 (−2.94, 1.94)	−0.43 (−2.77, 1.90)	0.35 (−0.87, 1.58)	-0.97 (−3.40, 1.47)
Employment type						
Temporary employee	1 (reference)	1 (reference)	1 (reference)	1 (reference)	1 (reference)	1 (reference)
Long-term employee	0.84 (−1.11, 2.79)	0.66 (−0.34, 1.65)	−1.98 (−4.03, 0.07)	1.26 (−0.77, 3.30)	0.60 (−0.46, 1.66)	−1.78 (−3.89, 0.33)
Years of experience (years)						
<5	1 (reference)	1 (reference)	1 (reference)	1 (reference)	1 (reference)	1 (reference)
5−10	0.31 (−1.71, 2.33)	0.53 (−0.53, 1.58)	−2.10 (−4.21, 0.01)	0.41 (−1.61, 2.42)	0.59 (−0.47, 1.64)	−2.06 (−4.16, 0.04)
11−20	0.55 (−1.72, 2.81)	0.27 (−0.92, 1.45)	−1.07 (−3.44, 1.30)	0.66 (−1.60, 2.91)	0.32 (−0.86, 1.51)	−0.98 (−3.33, 1.38)
≥21	0.10 (−2.40, 2.60)	−0.45 (−1.76, 0.86)	0.98 (−1.64, 3.60)	0.27 (−2.22, 2.76)	−0.40 (−1.71, 0.90)	1.07 (−1.53, 3.66)
Work role						
Prevention	1 (reference)	1 (reference)	1 (reference)	1 (reference)	1 (reference)	1 (reference)
Clinical	2.14 (0.56, 3.73) **	0.68 (−0.15, 1.50)	1.96 (0.31, 3.62) *	1.92 (0.34, 3.50) *	0.57 (−0.26, 1.40)	2.34 (0.69, 3.98) **
Clinical and prevention	1.31 (−0.40, 3.02)	0.81 (−0.08, 1.70)	0.71 (−1.08, 2.51)	1.22 (−0.48, 2.92)	0.70 (−0.19, 1.59)	0.93 (−0.84, 2.70)
Monthly salary						
<4000	1 (reference)	1 (reference)	1 (reference)	1 (reference)	1 (reference)	1 (reference)
≥4000	0.31 (−1.56, 2.18)	0.48 (−0.46, 1.42)	−1.20 (−3.17, 0.77)	0.52 (−1.49, 2.54)	0.38 (−0.68, 1.43)	−0.72 (−2.82, 1.37)
Perceived workload	5.30 (4.41, 6.18) ***	0.12 (−0.34, 0.58)	0.45 (−0.47, 1.37)	5.28 (4.39, 6.16) ***	0.10 (−0.36, 0.57)	0.51 (−0.42, 1.43)
Perceived work support	−1.72 (−2.43, −1.02) ***	−0.45 (−0.82, −0.08) *	2.23 (1.49, 2.97) ***	−1.73 (−2.44, −1.03) ***	−0.46 (−0.83, −0.09) *	2.27 (1.53, 3.01) ***
Perceived work autonomy	−0.68 (−1.33, −0.03) *	−0.42 (−0.76, −0.08) *	1.23 (0.55, 1.90) ***	−0.68 (−1.33, −0.03) *	−0.42 (−0.76, −0.07) *	1.21 (0.53, 1.89) ***
**Institution level**						
Workload				6.59 (3.46, 9.72) ***	0.20 (−1.31, 1.70)	1.47 (−1.43, 4.38)
Work support				−2.70 (−5.59, 0.20)	−0.15 (−1.54, 1.23)	3.49 (0.81, 6.17) *
Work autonomy				−0.47 (−3.74, 2.81)	−1.46 (−3.02, 0.10)	2.46 (−0.56, 5.48)
Country						
Country A				1 (reference)	1 (reference)	1 (reference)
Country B				−0.78 (−3.12, 1.57)	−0.63 (−1.75, 0.49)	3.95 (1.78, 6.11) ***
Country C				1.58 (−0.81, 3.98)	−0.54 (−1.70, 0.63)	3.38 (1.10, 5.66) **

* *p* value < 0.05, ** *p* value < 0.01, *** *p* value < 0.001.
